# Safety in primary care (SAP-C): a randomised, controlled feasibility study in two different healthcare systems

**DOI:** 10.1186/s12875-019-0909-8

**Published:** 2019-01-30

**Authors:** Caoimhe Madden, Sinéad Lydon, Margaret E. Cupples, Nigel D. Hart, Ciara Curran, Andrew W. Murphy, Paul O’Connor

**Affiliations:** 1 Department of General Practice, School of Medicine, National University of Ireland, Galway, Galway, Ireland; 2 HRB Primary Care Clinical Trial Network Ireland, National University of Ireland, Galway, Galway, Ireland; 3 School of Medicine, National University of Ireland, Galway, Galway, Ireland; 4 Irish Centre for Applied Patient Safety and Simulation, NUI Galway, Galway, Ireland; 5 Centre for Medical Education, School of Medicine, Dentistry and Biomedical Sciences, Queen’s University Belfast, Belfast, UK; 6 UK Clinical Research Collaboration Centre of Excellence for Public Health, Queen’s University Belfast, Grosvenor Road, Belfast, BT12 6BJ UK; 7 Centre for Public Health, School of Medicine, Dentristry and Biomedical Sciences, Queen’s University Belfast, Belfast, UK

**Keywords:** Patient safety, General practice, Primary care, Feasibility, Randomised controlled trial, safety climate, safety culture

## Abstract

**Background:**

Patient safety research is conducted predominantly in hospital settings, with a dearth of insight from primary care, despite suggestions that 2.2% of primary care consultations result in a patient safety incident. This study aimed to assess the feasibility of an intervention intended to improve patient safety in general practice.

**Methods:**

A randomised controlled feasibility study was conducted with general practices in the Republic of Ireland (*N* = 9) and Northern Ireland (*N* = 2), randomly assigned to the intervention (*N* = 5) or control (*N* = 6) group. The nine-month intervention consisted of: 1) repeated safety climate (SC) measurement (using GP-SafeQuest questionnaire) and feedback (comparative anonymised practice-level SC data), and 2) patient record reviews using a specialised trigger tool to identify instances of undetected patient harm. For control practices, SC was measured at baseline and study end only. The intervention’s perceived usefulness and feasibility were explored via an end-of-study questionnaire and semi-structured interviews.

**Results:**

Thirteen practices were invited; 11 participated; 10 completed the study. At baseline, 84.8% of intervention practice staff (39/46) and 77.8% (42/54) of control practice staff completed the SC questionnaire; at the study terminus, 78.3% (36/46) of intervention practice staff and 68.5% (37/54) of control practice staff did so. Changes in SC scores, indicating improvement, were observed among the intervention practices but not in the control group. The trigger tool was applied to 188 patient records; patient safety incidents of varying severity were detected in 19.1% (36/188). Overall, 59% of intervention practice team members completed the end-of-study questionnaire, with the majority in both healthcare systems responding positively about the intervention. Interviews (*N* = 9) identified the intervention’s usefulness in informing practice management and patient safety issues, time as a barrier to its use, and the value of group discussion of feedback.

**Conclusion:**

This feasibility study suggests that a definitive randomised controlled trial of the intervention is warranted. Our findings suggest that the intervention is feasible, useful, and sustainable. Practices were willing to be recruited into the study, response and retention rates were acceptable, and there is possible evidence of a positive effect of the intervention.

**Trial registration:**

The trial registration number is: ISRCTN11426121 (retrospectively registered 12th June 2018).

**Electronic supplementary material:**

The online version of this article (10.1186/s12875-019-0909-8) contains supplementary material, which is available to authorized users.

## Background

The importance of an increasing focus on patient safety in healthcare has been recognised [1]. However, whilst the majority of patient contacts occur in primary care [2], there has been a far greater focus on patient safety in hospital settings [2–5], arguably due to a perception of primary care as a relatively lower-risk endeavour [2]. However, both patient [6] and practice factors [7] have contributed to a growing complexity of clinical practice for general practitioners (GPs) which, combined with the sheer volume of patient contact, increases the potential for patient safety incidents (PSIs) in primary care. This is particularly concerning as GPs have reported several barriers to monitoring patient safety, such as limited and unreliable data on serious incidents, time to review practice-level data, and lack of examples of serious harm or ‘never’ events that are applicable to primary care settings [8].

Recognising the limited data on patient safety in primary care, the World Health Organisation has noted the pressing need to study and address patient safety in this setting [9]. In secondary care settings, targeted strategies have been implemented [10], with varying degrees of supporting evidence. However, a systematic review [3] of interventions to improve safety culture in primary care identified only two published interventional studies. Although both studies reported positive outcomes, methodological issues precluded the derivation of conclusive recommendations [3].

The Scottish Patient Safety Programme in Primary Care (SPSP-PC) [4] is one of the first comprehensive and coordinated attempts to improve patient safety in primary care. This programme has been implemented in 90% of Scottish general practices, with 83% reporting that it enabled them to make changes within their practice, resulting in safer, higher quality patient care [4]. GP feedback on the programme’s acceptability, feasibility, and utility has been predominantly positive [11]. However, its impact has not yet been evaluated independently of its developers, or assessed using a strong experimental design. Given the dearth of information on safety interventions in primary care, we therefore aimed to inform the design of a definitive randomised controlled trial of a primary care patient safety intervention.

We report the feasibility of conducting a randomised controlled trial of an intervention, developed previously by SPSP-PC, within primary care settings in two different healthcare systems; the Republic of Ireland (RoI) and Northern Ireland (NI). Following recommendations for good practice when designing pilot and feasibility studies [12], we aimed to evaluate rates of recruitment and retention of practices, response rates to questionnaires, completion of outcome measures, and participants’ perceptions of the intervention and effects of the intervention on safety climate.

## Methods

Our study protocol was published previously and provides a detailed overview of the study’s methodology [13] in accordance with CONSORT guidelines for pilot studies [14].

### Design

A randomised controlled design was used, whereby participating practices were assigned to either the intervention or control group by an external researcher using online randomisation software. Given the nature of the intervention, blinding of practice assignment among the researchers was not feasible.

### Ethical approval

Ethical approval for the study was received from the Irish College of General Practitioners’ Research Ethics Committee (no reference number, approved January 2016) and the Office for Research Ethics Committees of Northern Ireland (16/NI/0008, approved February 2016).

### Recruitment

Our sample size was pragmatic, aiming to include practices of diverse size and location, and from two different healthcare systems, in order to test the intervention’s feasibility in a range of settings. A purposeful sample of RoI practices was recruited through the Western Research Network (WestREN) [15], an Irish GP research network. Practices were stratified according to size (large (> 2 GP principals) or small (≤2 GP principals) and location (urban or rural). In the RoI, 13 practices were invited to participate, with 11 practices agreeing: two declined, due to the required time commitment. In NI, two practices of similar size and location (large; urban) were invited through the NI Clinical Research Network Primary Care Group [16]: both agreed to participate.

**Fig. 1:**
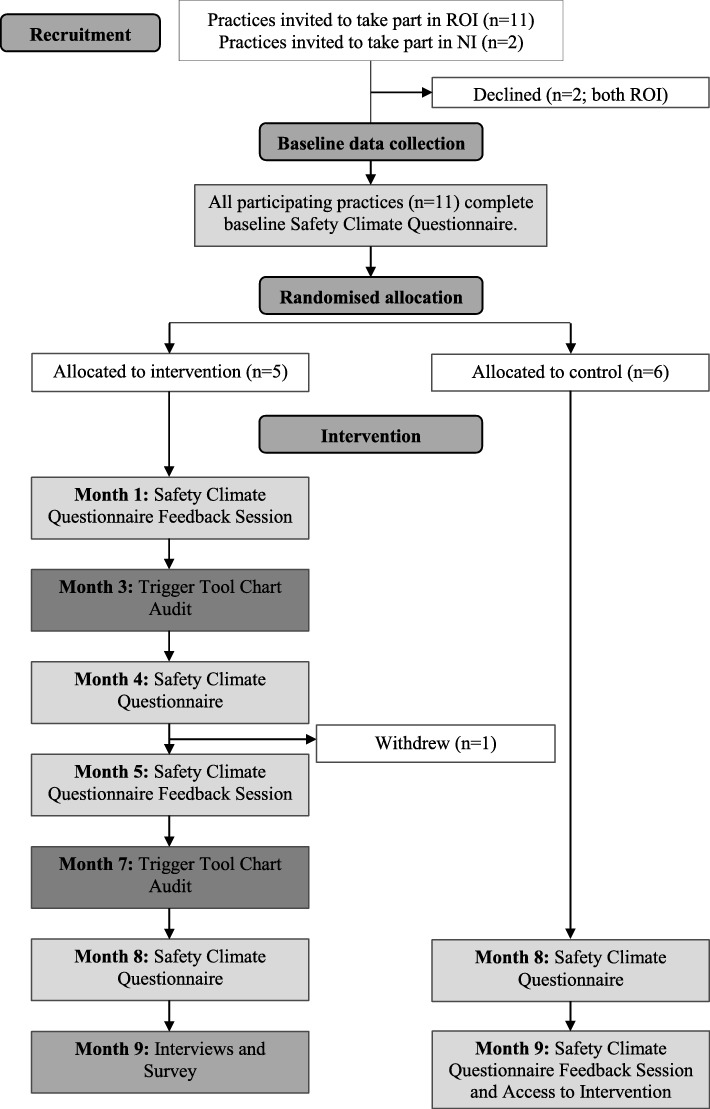
Flow diagram of the SAP-C feasibility study

Invitation letters were sent to the principal GP(s) in the selected practices (see Fig. 1) and, with their consent, other staff were then invited to participate. In the RoI, GPs provide services for private patients who pay for each consultation, and for patients with medical cards, whose healthcare is publicly funded. In NI, GP services are provided free-of-charge through the National Health Service.

### Setting and participants

Of the 11 practices who initially joined the study, 10 completed (8 RoI, 2 NI; 7 urban, 3 rural). A total of six of the practices were ‘large’. The mean number of managerial staff (GP principals, practice managers) was 4.8 (SD 1.5; range 3–7) and of non-managerial staff (non-principals, practice nurses, administrators) it was 7.5 (SD 2.9; range 4–11). Practices (*N* = 4) categorised as ‘small’ had a mean of 2 managerial staff (SD 0), and 4.3 (SD 1.7; range 2–6) non-managerial. In each practice, all staff provided personal written consent regarding their participation and all data were recorded anonymously.

### Procedure

#### Intervention practices

The intervention consisted of two components: (1) safety climate (SC) measurement and feedback and (2) patient record review using a specialised trigger tool to identify instances of harm (i.e., the trigger review method (TRM) [17]).

SC was assessed using the GP-SafeQuest for primary care [18]. SC is described as a measurable snapshot of an underlying safety culture at a particular period of time [3, 10, 19]. The GP-SafeQuest is a valid and reliable [20] survey instrument, designed specifically to measure SC perception, in primary care settings, across five subscales (leadership, teamwork, communication, workload, and safety systems), using Likert scales. Paper copies and stamped addressed envelopes were delivered to practices at three time points (baseline, study midpoint, and study terminus; see Fig. 1).

Each intervention practice received individualised practice-level feedback (a written report) on their baseline and study midpoint SC surveys, within a month of survey completion. Simple descriptive statistics and illustrative diagrams allowed comparison of SC scores with other practices’ anonymised data. Additionally, a research team member presented and led discussion of the findings at a practice meeting where the primary care team also discussed PSIs identified through reviews of their patients’ records, as described below.

One GP from each practice was asked to conduct a patient record review using the TRM at 3 and 7 months (see Fig. 1) [17]. The GP who conducted the review attended a two-hour training workshop delivered one-to-one by a facilitator (CC) using previously developed materials [11]. It is important to indicate that the TRM was an intervention component and not an outcome measure: its purpose was to facilitate the identification of specific patient safety issues within each practice.

At each time-point, the reviewer was requested to apply the TRM [17, 21] to a minimum of 20 and maximum of 30 records from a high-risk group (aged > 75 years), randomly selected from patients who had attended the practice during the previous three months. Records were first reviewed in order to detect whether they contained a ‘trigger’, defined as flags, occurrences or prompts that alert reviewers to potential errors and previously undetected adverse events (e.g., more than three consultations in seven days, hospital admission, repeat medication stopped) [17]. If a trigger was found, the record was reviewed in greater detail to determine whether the patient experienced any harm. Based on the definition used by De Wet and Bowie [17], harm was defined as ‘anything that happens as a result of interaction with health services that you would not want to happen to you or your relatives’. If no harm was detected, or the reviewer was unsure whether harm had occurred, they were advised not to record the incident. If harm was detected, the reviewer classified its perceived severity, and whether it was preventable and originated in secondary or primary care, using previously developed rating scales [11].

Participants completed a Trigger Review Summary Report (TRSR) [11], a standardised form containing a summary of anonymised data on the number of detected triggers, details of PSIs, and actions that were or should be taken as a result of the review.

Each intervention practice received 1000 Euro for participating. All participants from control and intervention practices were entered into a draw for a 100 Euro voucher at study baseline and terminus.

#### Control practices

All staff in control group practices were invited to complete the GP-SafeQuest [18] at baseline and at the study’s terminus (see Fig. 1). Feedback on their SC was provided at the conclusion of the study, with access to the intervention materials.

### Outcome measures

#### Safety climate

The GP-SafeQuest was used to evaluate the impact of the intervention, with SC measured at the beginning and end of the study in all practices (see Fig. 1).

#### Process evaluation

Process outcomes of interest were: willingness of practices to participate; response rates to questionnaires; retention of control and intervention practices; and the intervention group’s views on the feasibility, usefulness, and sustainability of the intervention. Semi-structured interviews were conducted (see ‘Interview Schedule for SAP-C’ in Additional files 1 and 2) with between one and three members of each intervention practice team regarding their perceptions of the intervention and an end-of-study questionnaire was circulated to all members of the intervention practices. Purposive sampling was used to select interview candidates, in order to obtain perspectives from a range of healthcare professionals and practices. We aimed to interview at least one GP in each practice and, where feasible, others with nursing or administrative responsibilities.

Interviews were conducted by a human factors psychologist (POC) and General Practitioner (MC). The interviews were either carried out by phone or face-to-face, digitally recorded and then transcribed.

#### Final feedback questionnaire

A feedback questionnaire was distributed for completion at the completion of the study (see ‘Safety in Primary Care (SAP-C) Feedback’ in Additional files 1 and 2).

### Data analysis

#### Safety climate questionnaire

Descriptive analysis was used [12] to report means and standard deviations of the five subscale scores and total scores on the GP-SafeQuest for each group at baseline and the study terminus. The effect size, regarding differences in pre-test and post-test subscale and total GP-SafeQuest scores, was computed.

#### Patient record review using TRM

Descriptive analysis was carried out on the number of records reviewed, triggers identified, number and type of PSIs recorded, PSI severity, preventability and origin, and changes made following the review.

#### Feedback questionnaire

Descriptive analysis was used to examine feedback questionnaire responses.

#### Interviews

Interview transcripts were analysed using the Framework Method [22] of thematic analysis which provided a structured process to summarise and explain the data. After two researchers independently coded three of the transcripts, a set of codes was agreed upon and grouped into clearly defined categories to form a working analytical framework which was then applied to all interviews by one researcher.

## Results

Thirteen practices were invited to participate, eleven accepted (85%), and one (intervention group) withdrew at month 4 (after first chart review) when the lead GP left the practice. Data from that practice were excluded from the analysis. The study ran from November 2016 to July 2017.

### Safety climate questionnaire

The SC questionnaire staff response rate was 84.8% (39/46) for intervention practices and 77.8% (42/54) for controls at baseline and 78.3% (36/46) for intervention practices and 68.5% (37/54) for controls at the study terminus. Overall, the questionnaire was completed by 81% (range 42.9–100%) and 73% (range 57.1–100%) of practice staff at baseline and study terminus respectively.

At baseline, 35.8% (29/81) of respondents were in managerial roles (GP principal, practice manager), and 61.7% (50/81) had non-managerial positions (GP assistant/ locum/ trainee/ intern, practice nurse, administrator, pharmacist). At the study terminus, 32.9% (24/73) of respondents were in managerial positions; 63% (46/73) were in non-managerial positions.

Table 1 shows means, standard deviations, and differences in subscale and total SC scores, before and after the intervention. Across the two time-points, total SC scores increased for the intervention group, suggesting an improved safety climate (negative Cohen’s d), but fell slightly in the control group. Effect sizes for the intervention group were generally small to moderate, with the strongest differences (Cohen’s d > 0.6) observed in the teamwork, safety systems and total SC scores of managerial staff.

**Table 1:** Means, standard deviations, and effect sizes of Safety Climate (SC) subscale and total scores calculated for control and intervention practices

	Safety Climate scores: Control practices						Safety Climate scores: Intervention practices					
	Managerial			Non-managerial			Managerial			Non-managerial		
	T1 Mean (SD)	T2 Mean (SD)	d^a^	T1 Mean (SD)	T2 Mean (SD)	d^a^	T1 Mean (SD)	T2 Mean (SD)	d^a^	T1 Mean (SD)	T2 Mean (SD)	d^a^
Workload	3.48 (0.93)	3.77 (1.34)	− 0.25	4.16 (1.37)	4.20 (1.63)	−0.02	4.70 (1.22)	4.88 (0.93)	−0.16	4.64 (1.09)	5.12 (1.06)	−0.45
Communication	5.78 (0.89)	5.07 (1.48)	0.58	5.03 (1.46)	5.05 (1.49)	−0.01	5.73 (0.97)	6.00 (0.77)	−0.31	5.47 (1.19)	5.78 (1.14)	−0.27
Leadership	6.24 (0.86)	6.13 (0.78)	0.13	5.90 (1.05)	5.68 (1.48)	0.17	6.29 (0.43)	6.51 (0.55)	−0.45	6.07 (0.86)	6.17 (1.16)	−0.10
Teamwork	5.62 (0.93)	5.66 (1.13)	−0.0	5.81 (0.96)	5.59 (1.04)	0.21	6.16 (0.61)	6.52 (0.56)	−0.61	6.09 (0.90)	6.16 (0.81)	−0.08
Safety systems	5.79 (0.65)	5.44 (0.95)	0.43	5.28 (1.13)	5.33 (1.27)	−0.04	5.87 (0.73)	6.40 (0.52)	−0.84	6.01 (0.89)	6.11 (0.83)	−0.12
Total SC	5.52 (0.63)	5.34 (0.91)	0.22	5.36 (0.91)	5.21 (1.09)	0.14	5.78 (0.57)	6.15 (0.55)	−0.66	5.70 (0.70)	5.94 (0.74)	−0.33

T1 = pre-test (baseline), T2 = post-test (end of study), d = Cohen’s d

^a^d represents the effect size of the difference between the scores at T1 and T2

### Patient record review using the TRM

Overall, 188 records were reviewed across the two chart reviews; triggers were identified in 150 (79.8%). In total, 36 PSIs were identified: 19.1% (36/188) of records reviewed contained a PSI.

Table 2 shows the severity and preventability of PSIs identified: 13.9% resulted in prolonged, substantial or permanent harm, including hospitalisation, and 27.8% were deemed to have been less severe, with the potential to cause harm. A total of 19.4% of PSIs were preventable and originated in primary care.

**Table 2:** Severity and preventability of patient safety incidents (*n* = 36) as identified by trigger tool

Rating scale	Description	n (%)
Severity		
1	Any incident with the potential to cause harm.	10 (27.8)
2	Mild harm, inconvenience, further follow-up or investigation to ensure no harm occurred.	13 (36.1)
3	Moderate harm: required intervention or duration for longer than a day.	8 (22.2)
4	Prolonged, substantial or permanent harm, including hospitalisation.	5 (13.9)
Preventability		
1	Not preventable and originated in secondary care.	2 (5.6)
2	Preventable and originated in secondary care OR not preventable and originated in primary care.	10 (27.8)
3	Potentially preventable and originated in primary care.	17 (47.2)
4	Preventable and originated in primary care.	7 (19.4)

Table 3 details the most commonly identified types of PSIs, the most frequent being related to medication and monitoring.

**Table 3:** Frequency and details of main types of patient safety incidents (n = 36) and the most common medications (*n* = 21) implicated

PSI Characteristics	n (%)
Types of PSIs^a^:	
Medication	21 (58.3%)
Monitoring	15 (41.7%)
Diagnosis and diagnosing	9 (25%)
Coding/record keeping	7 (19.4%)
Investigations	7 (19.4%)
Communication	6 (16.6%)
Unclear/insufficient info to classify	3 (8.3%)
Medications most commonly implicated in PSIs^b^:	
Diuretics	8 (38.1%)
ACEI/ARBs	3 (14.3%)
Opiates	3 (14.3%)
Antibiotics	2 (9.5%)
Warfarin	2 (9.5%)
Other hypoglycaemic agents	1 (4.8%)
NSAIDs including aspirin	1 (4.8%)
Not specified	3 (14.3%)

*Note.* ^a^Figures do not total to 36 as some PSIs fall within more than one of the categories

^b^Figures do not total to 21 as more than one medication was implicated in the instance of some PSIs

*ACEI* angiotensin-converting enzyme inhibitors, *ARB* angiotensin II receptor blockers, *NSAID* nonsteroidal anti-inflammatory drug

Various actions were taken immediately or planned by the intervention practices, based upon their TRM findings. Immediate actions related predominantly to improvements in coding/record keeping (e.g., adverse drug event code added), prescribing (e.g., repeat blood tests for patients with repeat prescription), communication (e.g., community level referral pathway clarified for diabetic patients), and investigations (e.g., recall for overdue bloods). The most common actions planned included feedback to colleagues (e.g., discussion of NSAID prescribing in renal impairment), management (e.g., more intensive monitoring of patients on repeat prescriptions), and updating or developing a protocol (e.g., updating warfarin prescribing protocol following ICGP guidelines).

### End-of-study questionnaire

Overall, we evaluated end-of-study questionnaires for 59% (27/46) of the intervention practice staff; the true response rate was probably higher as, due to an administrative error, some participants did not receive a questionnaire. Most respondents either agreed or strongly agreed that feedback on the SC survey and trigger tool chart audit was useful for improving patient safety (Table 4). Almost all (92.6%) agreed/strongly agreed that completing the survey helped them reflect on how patient safety was managed in practice.

**Table 4:** Intervention Practice responses (*N* = 27) to statements in end-of study questionnaire

	Strongly disagree	Disagree	Neither agree nor disagree	Agree	Strongly agree
	N (%)	N (%)	N (%)	N (%)	N (%)
Feedback on the safety climate survey was useful for improving patient safety.	1 (3.7%)	2 (7.4%)	1 (3.7%)	15 (55.6%)	8 (29.6%)
Completing the survey helped me reflect on how we manage patient safety in this practice.	1 (3.7%)	–	1 (3.7%)	16 (59.3%)	9 (33.3%)
Feedback from the trigger tool chart audit was useful for improving patient safety.	1 (3.7%)	–	6 (22.2%)	12 (44.4%)	8 (29.6%)
Changes were made at this practice based upon the information obtained from this intervention.	1 (3.7%)	6 (22.2%)	5 (18.5%)	10 (37%)	5 (18.5%)
Overall, I believe that this intervention had a positive effect on patient safety at this practice.	2 (7.4%)	3 (11.1%)	4 (14.8%)	11 (40.7%)	7 (25.9%)
The effect of this intervention is worth evaluating as a randomised controlled trial.	2 (7.4%)	–	5 (18.5%)	13 (48.1%)	7 (25.9%)

Approximately half of respondents (55.5%) agreed/strongly agreed that changes were made based upon information obtained from the intervention. Two thirds of respondents (66.6%) agreed/strongly agreed that it had a positive effect on patient safety, while 74% agreed/strongly agreed that it was worth evaluating as a randomised controlled trial.

Open-ended responses confirmed individuals’ Likert scale responses. A total of 19 of the respondents (70.4%) provided open ended responses. Written comments that the intervention allowed ‘*reflection on current practice*’ and ‘*prompted consideration of factors which put patients at higher risk*’, suggested that respondents had considered the questions in relation to their personal work situation. Concerns included whether ‘*it may engender negative feelings in practice whereby staff are not receptive to honesty*’, and that ‘*the volume of records [reviewed] were insufficient to make viable recommendations*’. In order to improve the intervention, participants suggested that practices should ‘*assign more time to partake in the study*’.

### Interviews

Nine members of the practice teams were interviewed across the four intervention practices, including: three principal GPs, three non-principal GPs, one practice nurse, and two administrators. Two interviews were carried out by telephone, and seven were conducted face-to-face. No invitees declined to be interviewed but not all who agreed were available at times convenient to the researcher and recruitment ceased when data saturation was considered to have been achieved. Interviews lasted approximately ten minutes. Three themes emerged from the analysis of interview data, with supporting quotes anonymised by the interviewee’s role (managerial/ non-managerial: M/ NM) and participant number.

#### Benefits from the intervention

Several interviewees considered that the intervention was helpful in raising awareness of safety issues, promoting learning and *“beneficial for your own practice…and for your own patients”* (NM1). One commented that the intervention “*would cover areas that I suppose we mightn’t, you know, be aware of”* (M1)*,* and noted that it “*highlighted things to us that we mightn’t have realised at all were issues”*.

The SC survey was beneficial in providing a ‘voice’ for more junior and non-managerial staff. Interviewees recognised that *“it’s hard to raise concerns on a personal basis…it’s good to do this sort of thing anonymously”* (NM6) and commented on the tailored practice-level feedback: *“it was good to see how you compared to other practices as well to use it as a benchmark”* (NM3). Group discussions identified different approaches within practices, of primary care team members, to decision-making and patient management, that were previously unrecognised. Revelations of “w*here unnecessary work was being done and where gaps were being left”* (NM4) were beneficial to organisational planning.

#### Changes and improvements in practice

One interviewee reported how the intervention had indicated that *‘changes should be made…….but that takes effort and time’* (NM4)*.* However, this perception of inaction was a deviant view: other interviewees, including others from the same practice, reported changes in practices’ processes and management. For example, *“note taking got a bit better after house calls”* (NM1), changes were made to protocols; “*we were all actually using different protocols kind of to change the Warfarin dosage…we standardised that and we have it in now in our system”* (NM3), *“the prophylactic antibiotics given for UTI… are now changed to nitrofurantoin – because guidelines had changed”* (NM6). The intervention encouraged clinical meetings in one practice and in another, there was a plan to *“try to make discussions at those more inclusive”* (NM5)*.*

#### Acceptability and recommendations

Most interviewees welcomed the intervention; one noted *“I definitely think it’s worth it”* (NM2), although a commonly perceived barrier was lack of time; *“It was time intensive- with current workloads and demands on time it couldn’t be given a priority”* (NM5)*,* and that *“many practices do not have the resources*” (M3).

The general consensus was that the intervention was worth investigating in a larger trial and that evidence of its effectiveness would promote its use in routine practice. Comments revealed perceptions that the SC questionnaire *“asked same basic questions in different ways”* (NM4) but this was welcomed as it allowed opportunity to consider different situations within their responses. Also, comments indicated some uncertainty in identifying PSIs: group discussion was valued in developing an agreed definition. Interviewees offered recommendations, such as, *“… the [TRM] tool needs a few tweaks- it’s a good way of assessing how things go but thought should be given to giving it more rigour”* (NM6) and, regarding the time required, *“obviously that has to be reimbursed”* (NM3).

## Discussion

This novel study investigated the feasibility of determining the impact of an intervention, derived from the SPSP-PC [4], on the SC of primary care practices in two different health care systems, one with a mixed public/private economy and the other funded publically. Within both these systems our findings regarding rates of recruitment, retention and completion, and evidence of potential positive impact suggest that a definitive trial is feasible. Only two practices declined to participate; one practice withdrew from the study, after the doctor leading participation left the practice, demonstrating the importance of a local ‘champion’ to drive the intervention. The overall response rates to the SC (over 70%) and end-of-study questionnaires (59%) were encouraging: obtaining good response rates from GPs is challenging [23].

Our intervention group’s perceptions that the TRM was useful in improving patient safety concurred with previous reports [4]. The number of PSIs, their severity, and the proportions considered preventable and originating in primary care were within the range of previous studies reporting TRM use in primary care; a recent systematic review reported an overall mean of 12.6 safety incidents (range: 2.3 to 26.5) per 100 records [24]. However, caution should be taken in comparing numbers of PSIs between studies as differences may be attributable to different methodologies. The chart review in our study was used to identify areas for improvement: its reliability in assessing PSIs as a measure of patient safety cannot be determined as it was conducted by only one GP, with limited training, in each practice.

The intervention was generally well received but time was the main barrier to its use. Therefore, as previously suggested [25], there would be a need to incentivise participation and compensate practices for the time required to participate in a definitive trial of the intervention. Such incentives could be financial, or alternatives may be identified by prospective trial participants. Particularly if the intervention is to be translated into routine practice, the issue of time allocation for its use must be considered.

In the control practices, changes in subscale and overall SC scores were either in the undesired direction or minimal but in intervention practices, all the changes in SC scores were in the desired direction, although the size of change varied across subscales. The mean scores we observed on the five SC subscales are broadly comparable to other studies which have used the GP-SafeQuest survey [26]. Concurring with previous reports, managerial groups tended to have a more positive view of SC than had the non-managerial groups [20, 27, 28]. Of note, amongst the intervention managerial group, the workload subscale had the lowest mean score [20, 26, 29]. High workload is of particular concern given that it has been found to have a negative effect on patient care [30] and doctor well-being [31], as well as being considered to be a major contributor to the recruitment and retention crisis facing UK general practice [32, 33]. There is a need for improved understanding of the contributors to workload in primary care and of its implications for patient safety.

Although it is widely agreed that a good SC is associated with safety, and that there is a relationship between safety culture and PSIs, the nature of the causal relationship between these variables is not well understood [34]. Therefore, whilst we have identified a potential positive effect on SC from our intervention, which may be used to inform a sample size calculation for a definitive trial, other appropriate primary outcome measures of patient safety should also be considered. One such measure may be the change in the number of PSIs [35] identified in an independent and blinded review of high-risk patient charts using the TRM. However, this outcome was not explicitly assessed in the current study and has limitations, particularly in terms of the required resources and recent European data protection regulations. These regulations require consent from patients for review of their records, or the records must be anonymised prior to review [36]. Another interventional technique developed by the SPSP-PC, the safety checklist for general practice [29], which offers a practical approach to the identification of potential hazards in the practice [37], may also be considered for use in further work. However, of note, a recent systematic review has identified that as yet there is no evident “best” method of measuring patient safety in primary care [38].

### Limitations and strengths

Consistent with recommendations that analysis of feasibility studies should be mainly descriptive, we did not conduct inferential statistics or hypothesis testing [11, 39, 40]. Our sample size was pragmatic, aiming to include practices of diverse size and location, and from two different healthcare systems. There was a potential for selection bias to occur, as practices in which there is a greater focus on patient safety, and consequently an increased motivation to implement interventions to improve patient safety, may be more likely to choose to partake in the proposed study and this may impact upon intervention outcomes. Whilst there was a reliance on self-report (i.e., of SC and PSIs) rather than objective data, a strength of our study was that we preserved anonymity of all data, both within and between participating practices in order to minimise reporting bias.

## Conclusion

We believe our feasibility study suggests that a novel definitive randomised controlled trial of an intervention to improve patient safety in primary care is warranted. Our findings suggest that the intervention we have tested is feasible, useful, and sustainable in two different healthcare systems but requires recognition of time required, particularly for reviewing records. There is evidence of a possible positive effect, practices were willing to be recruited, response rates were acceptable, and almost all participants remained for the duration of the trial. However, further consideration is required regarding the clinical significance of changes in SC scores and choice of an appropriate primary outcome measure.

## Additional files

Additional file 1: Interview Schedule for SAP-C. The list of questions used during the post-trial interviews. (DOCX 13 kb)

Additional file 2: Safety in Primary Care (SAP-C) Feedback. A feedback questionnaire distributed to all participants at the study end. (DOCX 17 kb)

## Abbreviations

GPs: General practitioners
ICGP: Irish College of General Practitioners
NI: Northern Ireland
PSIs: Patient safety incidents
RoI: Republic of Ireland
SC: Safety climate
SD: Standard deviation
SPSP-PC: Scottish Patient Safety Programme in Primary Care
TRM: Trigger review method
TRSR: Trigger review summary report
WestREN: Western Research Network
