# Supplementary material for: Safety in primary care (SAP-C): a randomised, controlled feasibility study in two different healthcare systems
Source: BMC Fam Pract. 2019 Jan 30;20:22. doi: 10.1186/s12875-019-0909-8 (PMC6352328; doi:10.1186/s12875-019-0909-8)
Supplement: Supplementary file 1 — Interview Schedule for SAP-C. The list of questions used during the post-trial interviews. (DOCX 13 kb) [file 12875_2019_909_MOESM1_ESM.docx]

**Interview Schedule for SAP-C**

1. What did you think about the usefulness of the feedback on the safety climate survey for improving patient safety?
2. What did you think about the usefulness of the feedback from the trigger tool chart audit?
3. Were any changes made at the practice based upon the feedback from the intervention, and if any were made what were they?
4. How do you think the intervention impacted patient safety at this practice?
5. What, if any, were the challenges to implementing this intervention?
6. What do you think of the intervention as a method for improving patient safety in primary care?
