# Supplementary material for: Safety in primary care (SAP-C): a randomised, controlled feasibility study in two different healthcare systems
Source: BMC Fam Pract. 2019 Jan 30;20:22. doi: 10.1186/s12875-019-0909-8 (PMC6352328; doi:10.1186/s12875-019-0909-8)
Supplement: Supplementary file 2 — Safety in Primary Care (SAP-C) Feedback. A feedback questionnaire distributed to all participants at the study end. (DOCX 17 kb) [file 12875_2019_909_MOESM2_ESM.docx]

| **Safety in Primary Care (SAP-C) Feedback**  **We would like to collect some final reactions on your thoughts about the usefulness of the SAP-C intervention. We would be grateful if you would answer the questions below.** |
| --- |

**1. Please circle the appropriate response.**

|  | *Strongly disagee* | *Disagree* | *Neither agree nor disagree* | *Agree* | *Strongly agree* |
| --- | --- | --- | --- | --- | --- |
| Feedback on the safety climate survey was useful for improving patient safety. | 1 | 2 | 3 | 4 | 5 |
| Completing the survey helped me reflect on how we manage patient safety in this practice | 1 | 2 | 3 | 4 | 5 |
| Feedback from the trigger tool chart audit was useful for improving patient safety. | 1 | 2 | 3 | 4 | 5 |
| Changes were made at this practice based upon the information obtained from this intervention. | 1 | 2 | 3 | 4 | 5 |
| Overall, I believe that this intervention had a positive effect on patient safety at this practice. | 1 | 2 | 3 | 4 | 5 |
| The effect of this intervention is worth evaluating as a randomized-controlled trial. | 1 | 2 | 3 | 4 | 5 |

**2. What did you think was most useful about this intervention?**

**3. What did you think was least useful about this intervention?**

**4. What could be done to improve the intervention?**
